# Sleep Behavior and Self-Reported Infertility: A Cross-Sectional Analysis Among U.S. Women

**DOI:** 10.3389/fendo.2022.818567

**Published:** 2022-05-10

**Authors:** Zhu Liang, Jianqiao Liu

**Affiliations:** ^1^Department of Obstetrics and Gynecology, Center for Reproductive Medicine, Guangdong Provincial Key Laboratory of Major Obstetric Diseases, The Third Affiliated Hospital of Guangzhou Medical University, Guangzhou, China; ^2^Key Laboratory for Reproductive Medicine of Guangdong Province, The Third Affiliated Hospital of Guangzhou Medical University, Guangzhou, China

**Keywords:** sleep behavior, bedtime, waketime, infertility, NHANES

## Abstract

**Objective:**

To investigate the associations between sleep behaviors and female infertility.

**Methods:**

We conducted a cross-sectional study composed of 2175 U.S. women 18-44 years of age from the National Health and Nutrition Examination Survey (NHANES) (2015-2018). Bedtime/waketime and sleep duration were extracted from the sleep disorder questionnaire. Self-reported infertility was defined as a binary variable based on the participants’ response to the question, “Have you ever attempted to become pregnant over a period of at least a year without becoming pregnant?”. Multivariate logistic regression analyses were done to explore the relationship between sleep behaviors and female infertility.

**Results:**

Bedtime (OR=1.24; 95% CI, 1.10-1.40, *P* = 0.001) and waketime (OR=1.14; 95% CI, 1.01-1.28, *P* = 0.037) were associated with infertility. Waketime of 08:00 was the inflection point, above which the probability of infertility increased rapidly (OR=1.41; 95% CI, 1.11-1.79, *P* = 0.004). Sleep-wake behavior was significantly associated with infertility (OR=1.34; 95% CI, 1.16-1.53, *P* < 0.001) and participants with early-bed/early-rise behavior had the lowest risk.

**Conclusions:**

Among U.S. women 18-44 years of age, bedtime and waketime were significantly linearly and non-linearly correlated with infertility, respectively. Early-bed/early-rise behavior was associated with the lowest infertility rate. Further study is needed because the timing of sleep behaviors are modifiable factors and could be a novel strategy to cope with infertility.

## Introduction

Infertility is defined as the failure of pregnancy within 12 months of unprotected intercourse (1). The infertility rate of couples ranges from 14% to 25% throughout the world (2). Although infertility is a global health issue, seldom modifiable risk factors have been found out.

As a main lifestyle behavior, sleep is regulated by human homeostasis and circadian rhythms. Meanwhile, reproductive hormones are also closely related to circadian rhythms and synchronized by the suprachiasmatic nucleus, of which rhythmicity is mediated by CLOCK genes (3). Several studies showed that CLOCK mutant mice did not exhibit luteinizing hormone surge (3) and follicle stimulating hormone surge (4). Thus, there is concern that circadian rhythm disturbance could lead to infertility. Several parameters of sleep have been shown to be associated with reproductive function (5). Previous epidemiological studies showed that short sleep (<5-6 hours) could be associated with irregular menstrual cycles (6, 7), lower fecundability (8) or poorer *in vitro* fertilization (IVF) outcomes (9).

Apart from sleep duration, sleep behaviors (including bedtime and waketime) have also been linked to several health outcomes, such as diabetes, obesity and fatty liver disease (10–12). There were numerous studies showing that the circadian timing system is deeply integrated in female reproductive physiology, including neuroendocrine control of pituitary hormone secretion, gonadal steroid hormone biosynthesis and secretion, ovulation (13, 14). Circadian rhythms disruption exerted negative impacts on female reproductive function (5, 15). However, most of the previous studies focused on the impacts of sleep duration and shift work on fertility. Little is known about the association between sleep behaviors and female fertility. Theoretically, compared to those with early bedtime, people with late bedtime could have more light exposure at night (16, 17). It could potentially disturb circadian rhythms and subsequently cause abnormal secretion of reproductive hormones (18–20), leading to female infertility. Given that both bedtime and waketime are modifiable behaviors, it would have potential significant impact on public health if the associations between sleep behaviors and female infertility were found.

The aims of this study were (1) to investigate the relationship between sleep behaviors (including bedtime and waketime) and female infertility and (2) to figure out which type of sleep-wake behavior was associated with the lowest infertility rate, based on a large national population-based representative survey.

## Methods

### Data Source and Sample

The data of this study was extracted from two continuous cycles (2015-2016 and 2017-2018) of national population-based representative survey (NHANES), since only these two cycles included self-reported sleep questionnaire. NHANES is a cross-sectional survey administered by the National Center for Health Statistics (NCHS) of the Centers for Disease Control and Prevention (CDC) that collects data on the health and nutritional status of non-institutionalized civilian residents and conducts more detailed laboratory analyses on a subset of the participants. The survey was approved by the NCHS research ethics review board. Informed consent has been obtained from all participants.

**Figure 1:**
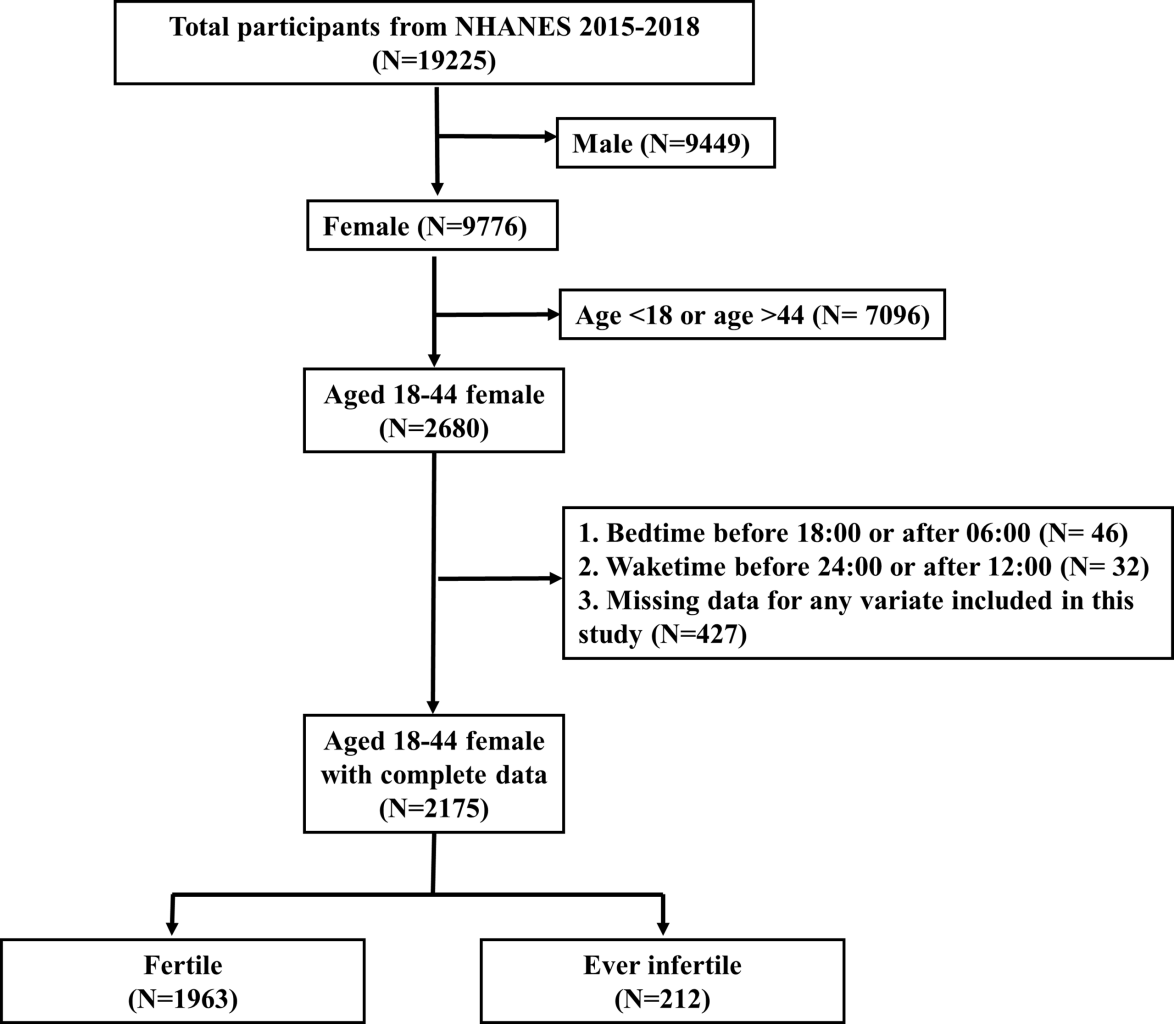
Flowchart of participant selection. NHANES, National Health and Nutrition Examination Survey.

There were 19225 participants in these two cycles. After excluding male (N=9449), age <18 or >44 (N=7096), bedtime before 18:00 or after 06:00 (N=46), waketime before 24:00 or after 12:00 (N=32) and missing data for any variate included in this study (N=427), there were 2175 participants included for analyses (**Figure 1**). We excluded participants with bedtime before 18:00 or after 06:00, or with waketime before 24:00 or after 12:00, because individuals with extreme sleep/wake behaviors might have night work schedule or other unhealthy lifestyle components, which could increase complexity.

Self-reported infertility was extracted from the reproduction health questionnaire. It was defined as a binary variable based on the participants’ response to the question, “Have you ever attempted to become pregnant over a period of at least a year without becoming pregnant?” (Variable Name in NHANES: RHQ074) Those who responded yes were considered ever infertile (1= ever infertile, 0= fertile).

### Independent Variables

Bedtime/waketime and sleep duration were extracted from the sleep disorder questionnaire. Sleep duration was extracted from the response to the question “Sleep hours”. For 2015-2016 cycle, bedtime and waketime were defined based on the participants’ response to the questions, “Usual sleep time on weekdays or workdays” (SLQ300) and “Usual waketime on weekdays or workdays” (SLQ310), respectively. For 2017-2018 cycle, bedtime was calculated by the formula: bedtime= (5 × sleep time on weekdays + 2 × sleep time on weekends)/7. “Sleep time on weekdays” and “sleep time on weekends” were defined according to the response to the questions, “Usual sleep time on weekdays or workdays” (SLQ300) and “Usual sleep time on weekends” (SLQ320), respectively. Similarly, “Wake time on weekdays” and “wake time on weekends” were defined according to the response to the questions, “Usual wake time on weekdays or workdays” (SLQ310) and “Usual wake time on weekends” (SLQ330), respectively. Subsequently, waketime was calculated by the formula: waketime= (5 × wake time on weekdays + 2 × wake time on weekends)/7.

### Covariates

Covariates were selected according to previous studies (1, 21), including age (RIDAGEYR), body mass index (BMI) (BMXBMI), poverty level index (INDFMMPI), physical activity (PAQ620), education (DMDEDUC2), race (RIDRETH1), marital status (DMDMARTL) and cotinine (LBXCOT). Poverty level index was defined as a ratio of monthly family income to the poverty guidelines specific to family size. The serum cotinine level was used to decide the smoking status (participants with cotinine of 3 ng/mL or higher were considered as a smoker) (22).

### Statistical Analyses

Continuous variables were reported as mean and standard deviation (SD) if they had normal distribution. Otherwise, they were presented as median (Quartile1 – Quartile 3; Q1-Q3). Categorical variables were described as whole numbers and percentages. Student’s *T* test or Mann–Whitney *U* test were used to analyze continuous variables according to their distribution. Fisher’s exact test or Chi-squared test were used to analyze categorical variables according to their frequencies.

Univariate and multivariate logistic regression analyses were applied to assess the linking of sleep behaviors (bedtime and waketime) to infertility. Multicollinearity in regression analyses was detected by variance inflation factor (VIF), with a reference value of 5. Since each variable’s VIF was lower than 5, all variables were selected into the multivariate analysis, including age, race, BMI, education, marital status, physical activity, poverty level index, cotinine, sleep duration and the independent variable (bedtime, wake time or sleep behavior).

In order to assess whether the independent variable in our study were robust risk factors in different models, we also employed Akaike information criterion (AIC) through a backward stepdown process to choose risk factors from the full multivariate regression model for each independent variable (bedtime, wake time or sleep behavior). AIC is a common criterion to select the fewest possible independent variables which could explain the greatest amount of variation. Therefore, if the independent variables were significantly associated with infertility in the full as well as in the AIC-based multivariate logistic regression, it would indicate the independent variables were robust risk factors for infertility, respectively.

To visualize the relationship between sleep behaviors and infertility, spline smoothing plots which were based on generalized additive model (GAM) were used to identify whether there were linear or non-linear relationships (23). Once there was a non-linear relationship, a two-piecewise linear regression model would be performed to figure out the optimal cutoff point for the independent variable. Specifically, a recursive method automatically calculated the inflection point, where the largest log-likelihood value in the two-piecewise linear regression model was reached (24, 25). Then the cutoff point was selected to dichotomize the independent variable.

After dichotomizing bedtime and waketime into categorical variables, participants were classified into one of four categories according to their bedtime and waketime combination: early-bed/early-rise (EE); early-bed/late-rise (EL); late-bed/early-rise (LE); late-bed/late-rise (LE). We compared the adjusted odds ratio among different categories to assess whether there were significant impacts of different sleep-wake behaviors on infertility.

We chose not to use sampling weights in our analyses, because when variables employed in the calculation of sampling weights are also included in statistical models, weighted analyses will lead to lowered accuracy of effect estimates (26, 27). Age, race and income were used in the calculation of sampling weights in NHANES. Meanwhile, we also included age, race and income as covariates in this study. Therefore, unweighted analyses were more suitable. To assess the robustness of our findings, we also assess the relationship between sleep behaviors and infertility with weighted samples. According to the guidance of NHANES, survey weighting from each 2-year cycle was combined to create a 4-year survey weight. Since some of the included variables were collected in the Mobile Exam Center (MEC) exam, we chose MEC weights in this study. The population represented in our study was calculated using the “survey” package according to NHANES weighting tutorials (https://wwwn.cdc.gov/nchs/nhanes/tutorials/Module3.aspx).

All statistical analyses were performed through R software version 3.4.2 (Institute for Statistics and Mathematics, Vienna, Austria; https://www.r-project.org/). The used R packages were “mice” for descriptive analyses, “Hmisc” for two-piecewise linear regression models, “mgcv” and “gdata” for generalized additive models and “survey” for weighted analyses. The “glm” function was also employed for logistic regression analyses. A two-tailed *P*-value <0.05 was considered to be statistically significant.

## Results

**Figure 1** showed the selection process for the study participants. After selection, there were 2175 eligible participants included for analyses, representing a population of 49,244,467 in the US. 1963 participants (90.25%; representing a population of 44,132,595) were fertile and 212 participants (9.75%; representing a population of 5,111,872) were ever infertile.

**Table 1** displayed the difference of baseline characteristics of selected participants. For unweighted analyses, participants with infertility were more likely to be older at the time of survey (34.13 years vs. 30.39 years, *P* < 0.001), have higher BMI (32.02 kg/m^2^ vs. 30.39 kg/m^2^, *P* < 0.001) and have smoking (29.06% vs. 21.58%, *P* = 0.015). With regard to the sleep-related variates, participants with infertility have significantly later bedtime (23:10 vs. 22:57, *P* = 0.037) and shorter sleep duration (7.84 hours vs. 8.04 hours, *P* = 0.022). There was a similar wake time between infertile and fertile participants (06:50 vs. 06:55, *P* = 0.467). For weighted analyses, there were significant differences between fertile group and ever infertile group for the following variables: BMI (29.66 kg/m^2^ vs. 33.77 kg/m^2^, *P* = 0.004) and marital status (Married/Living with partner: 63.98% vs. 75.52%, *P* = 0.029).

**Table 1:** Baseline characteristics of selected participants from the NHANES 2015-2018 (n = 2175; weighted sample, N = 49,244,467).

	Unweighted			Weighted		
Characteristic	Fertile	Ever infertile	*P*-value	Fertile	Ever infertile	*P*-value
	n = 1963	n = 212		N = 44,132,595	N = 5,111,872	
**Bedtime**	22:57 (1:28)	23:10 (1:28)	**0.037**	22:56 (22:47, 23:04)	23:59 (22:42, 23:16)	0.745
**Waketime**	6:55 (1:36)	06:50 (1:42)	0.467	6:50 (6:40, 6:59)	6:45 (6:28, 7:03)	0.675
**Sleep duration (hours)**	8.04 (1.41)	7.84 (1.60)	**0.022**	7.94 (7.84, 8.04)	7.79 (7.53, 8.04)	0.294
**Age (years)**	30.39 (8.06)	34.13 (6.69)	**<0.001**	31.11 (30.45, 31.77)	33.49 (31.50, 35.49)	0.051
**BMI (kg/m^2^)**	29.23 (8.31)	32.02 (8.61)	**<0.001**	29.65 (28.79, 30.50)	33.77 (31.20, 36.34)	**0.004**
**Poverty level index (%)**			0.121			0.448
≤ 1.30	40.02	40.41		31.64 (26.15,37.69)	39.12 (27.02,52.73)	
1.30 - ≤ 1.85	16.28	10.88		14.22 (11.48,17.48)	12.60 (6.57,22.8 1)	
> 1.85	43.70	48.70		54.15 (48.66,59.53)	48.28 (34.82,62.00)	
**Physical activity (%)**			0.708			0.693
Yes	40.65	41.98		47.85 (41.95,53.81)	51.00 (35.33,66.48)	
No	59.35	58.02		52.15 (46.19,58.05)	49.00 (33.52,64.67)	
**Education (%)**			0.665			0.632
Less than high school	15.01	14.49		10.14 (7.04,14.40)	13.77 (7.30,24.48)	
High school	19.11	21.74		21.33 (17.02,26.38)	19.91 (11.20,32.89)	
More than high school	65.88	63.77		68.53 (62.57,73.93)	66.32 (53.94,76.80)	
**Race (%)**			0.375			0.501
Mexican American	18.19	20.28		10.78 (7.19,15.86)	12.74 (6.07,24.82)	
Other Hispanic	11.16	9.43		8.35 (6.10,11.33)	4.86 (1.95,11.61)	
Non- Hispanic White	29.90	33.96		55.86 (48.96,62.54)	60.33 (47.73,71.69)	
Non- Hispanic Black	22.47	17.45		12.64 (8.94,17.58)	8.86 (5.63,13.69)	
Other Race	18.29	18.87		12.37 (9.83,15.47)	13.21 (6.86,23.90)	
**Marital status (%)**			**0.003**			**0.029**
Married/Living with partner	64.51	74.41		63.98 (57.91,69.63)	75.52 (63.14,84.75)	
Widowed/Divorced/Separated	15.54	15.17		13.20 (10.26,16.83)	15.90 (8.26,28.43)	
Never Married	19.95	10.43		22.82 (18.03,28.43)	8.58 (4.18,16.80)	
**Cotinine (%)**			**0.015**			0.881
< 3 (ng/ml) (non-smoker)	78.42	70.94		76.10 (70.60,80.86)	76.94 (63.92,86.27)	
≥ 3 (ng/ml) (smoker)	21.58	29.06		23.90 (19.14,29.40)	23.06 (13.73,36.08)	

1. BMI, body mass index.

2. Data in the table: (Unweighted analyses) For continuous variables: mean (SD), P-value was by Student’s T test if they had normal distribution; median (Q1-Q3), P-value was by Mann–Whitney U test if they were non-normalvariables. For categorical variables: (%), P-value was by Chi-square test.

(Weighted analyses) For continuous variables: survey-weighted mean (95% CI), P-value was by survey-weighted linear regression (svyglm); For categorical variables: survey-weighted percentage (95% CI), P-value was by survey-weighted Chi-square test (svytable).

*Mann–Whitney U test for continuous variables.

The bold values indicate a P-value <0.05.

Multivariate logistic regression analyses for infertility were displayed in **Tables S1, S2**, in terms of bedtime and wake time, respectively. **Table S1** showed that bedtime was significantly associated with infertility in the full multivariate logistic regression (OR, 1.24; 95% CI, 1.10-1.40, *P* < 0.001). After AIC criterion’s selection, bedtime remained to be a significant risk factor for infertility (OR, 1.19; 95% CI, 1.07-1.33, *P* = 0.001) (**Table S1**). Similarly, wake time was significantly associated with infertility in the full multivariate logistic regression (OR, 1.14; 95% CI, 1.01-1.28, *P* = 0.037), as well as in the AIC-based multivariate logistic regression (OR, 1.13; 95% CI, 1.00-1.27, *P* = 0.042) (**Table S2**). The results were consistent in the full or AIC-based multivariate logistic regression models, for bedtime and wake time, respectively. Thus, we chose to employed full multivariate logistic regression in the following analyses, since the results for bedtime and wake were consistent in both full and AIC-based multivariate models, which indicating sleep behavior (bedtime and waketime) were robust risk factors for infertility.

The relationship between sleep behaviors and infertility was shown in **Table 2**. All variables were included for multivariate analysis because none of the VIF of was > 5. Both bedtime and waketime were significantly associated with infertility in their continuous types (bedtime: adjusted odds ratio (OR), 1.24; 95% confidence interval (CI), 1.10-1.40; *P* = 0.001; waketime: adjusted OR, 1.14; 95% CI, 1.01-1.28, *P* = 0.037). In addition, bedtime and waketime in quartiles types were also significantly associated with the risk of infertility in multivariate-adjusted models (*P* = 0.001 and *P* = 0.037, respectively).

**Table 2:** Relationship between sleep behaviors (bedtime and waketime) and infertility in unweighted sample.

Exposure	Unadjusted		Adjusted*	
	OR (95%CI)	*P*-value	OR (95%CI)	*P*-value
**Bedtime (continuous)**	1.11 (1.01, 1.21)	**0.037**	1.24 (1.10, 1.40)	**0.001**
**Bedtime (quartiles)**				
18:00 - < 22:00	Reference		Reference	
22:00 - < 22:30	1.03 (0.62, 1.69)	0.918	1.17 (0.66, 2.05)	0.591
22:30 - < 23:00	0.58 (0.29, 1.17)	0.129	0.68 (0.31, 1.53)	0.356
23:00 - < 00:00	1.47 (0.94, 2.29)	0.088	2.04 (1.22, 3.42)	**0.007**
00:00 – 06:00	1.40 (0.90, 2.18)	0.136	2.22 (1.28, 3.85)	**0.004**
*P* for trend		**0.045**		**0.001**
**Waketime (continuous)**	0.97 (0.89, 1.06)	0.467	1.14 (1.01, 1.28)	**0.037**
**Waketime (quartiles)**				
00:00 - < 05:42	Reference		Reference	
05:42 - < 06:30	1.05 (0.67, 1.63)	0.840	1.08 (0.66, 1.76)	0.765
06:30 - < 07:00	1.12 (0.70, 1.78)	0.647	1.32 (0.78, 2.23)	0.302
07:00 - < 08:00	0.74 (0.47, 1.16)	0.184	1.07 (0.64, 1.81)	0.788
08:00 – 12:00	0.90 (0.59, 1.39)	0.643	1.79 (1.03, 3.10)	**0.038**
*P* for trend		0.409		**0.036**

OR, odds ratio; BMI, body mass index.

*Adjust for: age; race; BMI; education; marital status; physical activity; poverty level index; cotinine; sleep duration.

The bold values indicate a P-value <0.05.

**Figure 2:**
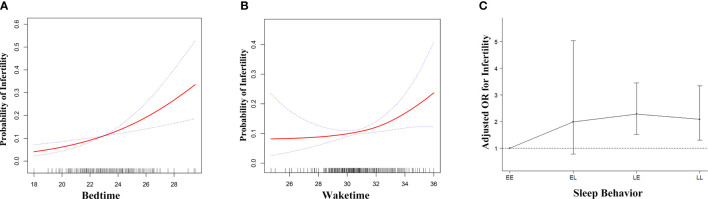
Adjusted associations of bedtime **(A)**, waketime **(B)** and sleep-wake behavior **(C)** with infertility in unweighted sample, respectively. While there was a positive linear association between bedtime and fertility **(A)**, a threshold, nonlinear association between waketime and fertility **(B)** was found in generalized additive models (GAM). Solid red line represents the smooth curve fit between variables. Blue bands represent the 95% of confidence interval from the fit. Each of the three independent variables, bedtime **(A)**, waketime **(B)** and sleep-wake behavior **(C)** was adjusted for age, race, BMI, education, marital status, physical activity, poverty level index, cotinine, bedtime, waketime and sleep duration except for the variable itself. EE, Early-bed/Early-rise; EL, Early-bed/Late-rise; LE, Late-bed/Early-rise; LL, Late-bed/Late-rise; OR, odds ratio; BMI, body mass index.

Generalized additive model was used to visualize the relationship between sleep behaviors and infertility (**Figure 2**). For bedtime, there was a positive linear relationship with infertility (**Figure 2A**), indicating that the probability of infertility significantly increased as bedtime became later. As for waketime, when waketime was between 00:00 and 08:00, the probability of infertility almost plateaued. When waketime was after 08:00, the probability of infertility increased rapidly as waketime became later (**Figure 2B**). Subsequently, a piece-wise linear regression was applied to select the inflection point for waketime. The result showed that 08:00 was the inflection point for waketime after adjusted-multivariate analysis (**Table S3**), since the logarithmic likelihood ratio test between wake time of 00:00 – < 08:00 and 08:00 - 12:00 was statistically significant (***P* = 0.043**). Specifically, when waketime was between 00:00 and 08:00, waketime was not significantly associated with infertility (adjusted OR, 1.00; 95% CI, 0.85-1.19, *P* = 0.970). However, when waketime was between 08:00 and 12:00, the risk of infertility increased significantly by 41% (*P* = 0.004) as waketime increase per one unit (hour).

After dichotomizing bedtime and waketime into categorical variables, **Table S4** showed that sleep behavior was significantly associated with infertility in the full multivariate logistic regression (adjusted OR for EL, 2.02, *P* = 0.137; for LE, 2.29, *P* < 0.001; for LL, 2.14, *P* = 0.001; *P* for trend < 0.001), as well as in the AIC-based multivariate logistic regression (OR for EL, 2.12, *P* = 0.074; for LE, 2.11, *P* < 0.001; for LL, 2.09, *P* = 0.002). Subsequently, we chose full multivariate logistic regression in the following analyses, since the results for sleep behavior were consistent in both full and AIC-based multivariate models, which indicating sleep behavior was a robust risk factor for infertility.

**Figure 2C** showed the association of bedtime-waketime categories and infertility. Sleep-wake behavior was significantly associated with infertility (adjusted OR=1.34; 95% CI, 1.16-1.53, *P* < 0.001). Among the four categories, participants in the EE group had the lowest probability of infertility. Compared to those who were in EE group, participants in EL, LE and LL group had higher probability of infertility (adjusted OR for EL, 2.02, *P* = 0.137; for LE, 2.29, *P* < 0.001; for LL, 2.14, *P* = 0.001; *P* for trend < 0.001) (**Table S5**).

## Discussion

In this study, we found that among U.S. women 18-44 years of age, both bedtime and waketime were significantly associated with infertility (bedtime: OR, 1.24; 95% CI, 1.10-1.40; *P* = 0.001; waketime: OR, 1.14; 95% CI, 1.01-1.28, *P* = 0.037). While bedtime had a positive linear relationship with infertility, there was a non-linear relationship between waketime and infertility. Waketime of 08:00 was the inflection point, above which the probability of infertility increased rapidly (*P* = 0.004). By combining bedtime and waketime into four categories, we further demonstrated that participants with EE type of sleep-wake behavior had the lowest infertility rate (*P* < 0.001). These findings indicated that sleep-wake behavior could be a potential modifiable behavioral factor for female infertility, providing a novel strategy to cope with infertility.

A previous study has shown that chronotype, which is defined as individual differences in sleep-wake cycles leading to the onset of distinct behavioral phenotypes, was related to the reproductive function in women (28). However, it did not record the precise sleep-wake behavior of each participant and therefore, it didn’t figure out the specific relationship between sleep behaviors (bedtime and waketime) and infertility. In addition, sleep duration was not included in that study, which could be a potential confounder since sleep duration was shown to be associated with reproductive function (15). Consistent with a previous study (15), there was a *U*-shaped association between sleep duration and female infertility (**Figure S1**). Sleep duration of 8.5 hours had the significantly lowest infertility risk. Therefore, we had adjusted sleep duration in the multivariate analysis. Our results suggested that sleep-wake behavior was significantly associated with female fertility, independent of sleep duration.

The variance in chronotype throughout the population follows a normal distribution (29). Sleep timing is largely regulated by the circadian clock (together with the sleep homeostat), and a conventional sleep period generally starts from 11 p.m. to 7 a.m (30). In our study, the average bedtime and waketime was 22:57 and 6:55 in fertile group, 23:10 and 06:50 in ever infertile group, which is consistent with previous studies. Currently, there are no recommendation on bedtime and waketime for women of child-bearing age. The National Sleep Foundation recommends an appropriate sleep duration of 7 to 9 hours for young adults and adults without indicating a specific sleeping time (31). However, evening chronotype was associated with a worse metabolic risk profile and mental health (32), which closely related to reproductive health. Our results may further suggest that not only sleep duration but also sleep behaviors are important in women of child-bearing age, and the cut-off points obtained through our statistics might provide a reference for future research.

One of the mechanisms underlying the relationship between sleep behaviors and infertility could be exposure to environmental cues at night. Staying up late is related to more light exposure at night, which could suppress the secretion of melatonin and subsequently disturb the circadian rhythms. In addition, melatonin has been shown to capture free radicals in oocytes (33) and therefore, the decreased melatonin secretion could lead to excessive oxidative stress in oocytes, causing premature ovarian failure. Another possible mechanism could be the genetic dysregulation of biological clock, since the CLOCK gene was shown to play an important role between circadian disruption and infertility (34).

Our study has several strengths. Firstly, the data were extracted from a large national population-based representative survey. Secondly, we used smoothing plots to visualize the relationship between sleep behaviors and infertility. Thirdly, we employed weighted samples to assess the robustness of our findings, and the results were shown to be consistent with the unweighted one (**Table S6** and **Figure S2**). Our findings have several important clinical implications. For women who are planning for pregnancy, it could provide helpful information to adjust their timetable of sleeping and waking. For reproductive endocrinologists, they could give patients advice on daily timetable, adding a complementary strategy to existing approaches to manage infertility.

In addition, the association of sleep behavior with infertility carries important public health implications. The World Health Organization recognizes infertility as a disease (35), which is estimated to affect 14% to 25% couples throughout the world. Although infertility is a global health issue, seldom modifiable risk factors have been found out. Given that both bedtime and waketime are modifiable behaviors, sleep behavior could be a potential target for public health campaigns. It is conceivable that a better sleep behavior may be associated with lower economic and human costs from infertility. Therefore, when making sleep health policy, it is also necessary to focus on sleep behavior, rather than merely concerning sleep disorders. In addition, previous sleep health education mainly focused on the student populations (36, 37). Our findings suggest that women who are planning for pregnancy should also receive sleep health education, in order to improve the quality of reproductive health.

However, our study does have several limitations. Firstly, due to the methodological features of NHANES, the data reflects participants’ characteristics which documented at the time of survey, rather than at the time of experiencing infertility. To minimize the impact of this limitation, in this study we excluded women older than 44, who might have large difference between the time of survey and the time of experiencing infertility. Secondly, owing to the inherent limitations of cross-sectional surveys, we cannot figure out the temporality of the occurrence of infertility and the occurrence of sleep behaviors. Thirdly, the timings of sleep behaviors were extracted from self-report questionnaires. Employing objective estimates of bedtime and waketime in future study could provide more precise estimates of sleep behaviors. Fourthly, due to the lack of information of sleep behaviors among cohabitants, such as partner or children, in this survey, we cannot rule out the influence of cohabitants on their sleep behaviors. Fifthly, we did not include laboratory tests in the logistic regressions due to their high frequency of missing data (> 50%). In the future, we will carry out prospective cohort study with employing laboratory tests, to validate the findings in this study. Sixthly, our conclusions are limited by an incomplete knowledge of participants’ history of pregnancy attempts. In future studies, we will pay attention to this problem and adopt more rigorous questioning methods to collect answers so as to obtain more reliable research results.

## Conclusions

In conclusion, we found that among U.S. women 18-44 years of age, bedtime and waketime were significantly linearly and non-linearly correlated with infertility, respectively. Early-bedtime/early-waketime was associated with the lowest infertility rate. Further study is needed because the timing of sleep behaviors are modifiable factors and could be a novel strategy to cope with infertility.

## Data Availability Statement

The datasets presented in this study can be found in online repositories. The names of the repository/repositories and accession number(s) can be found below: https://www.cdc.gov/nchs/nhanes/index.htm.

## Ethics Statement

The studies involving human participants were reviewed and approved by the NCHS research ethics review board. The patients/participants provided their written informed consent to participate in this study.

## Author Contributions

ZL conceived and designed the study. JL supervised the study and was responsible for literature review. ZL acquired and analyzed the data and did the statistical analysis. JL was contributed to the critical revision of the manuscript. The corresponding author attests that all listed authors meet authorship criteria. All authors accept responsibility for the paper as published.

## Funding

This research and researchers were funded as follows: The Medical Key Discipline of Guangzhou (2021-2023); The General Program of National Natural Science Foundation of China, grant no. 81971452; The National Key Research and Development Program of China, grant no. 2018YFC1003803.

## Conflict of Interest

The authors declare that the research was conducted in the absence of any commercial or financial relationships that could be construed as a potential conflict of interest.

## Publisher’s Note

All claims expressed in this article are solely those of the authors and do not necessarily represent those of their affiliated organizations, or those of the publisher, the editors and the reviewers. Any product that may be evaluated in this article, or claim that may be made by its manufacturer, is not guaranteed or endorsed by the publisher.
