# Supplementary figures and images for: Sleep Behavior and Self-Reported Infertility: A Cross-Sectional Analysis Among U.S. Women
Source: Front Endocrinol (Lausanne). 2022 May 10;13:818567. doi: 10.3389/fendo.2022.818567 (PMC9127231; doi:10.3389/fendo.2022.818567)

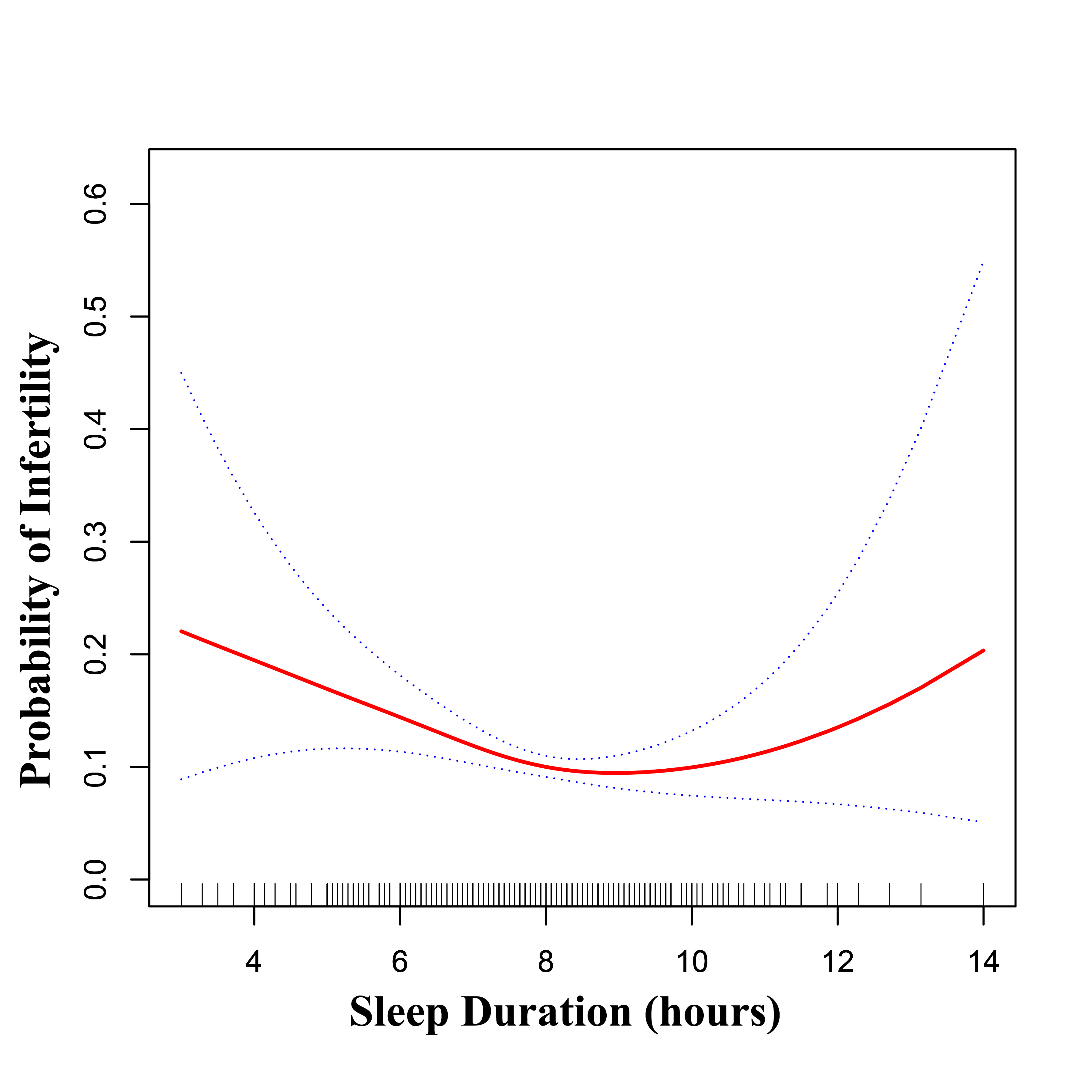

Supplement: Supplementary file 1 [file Image_1.tif]

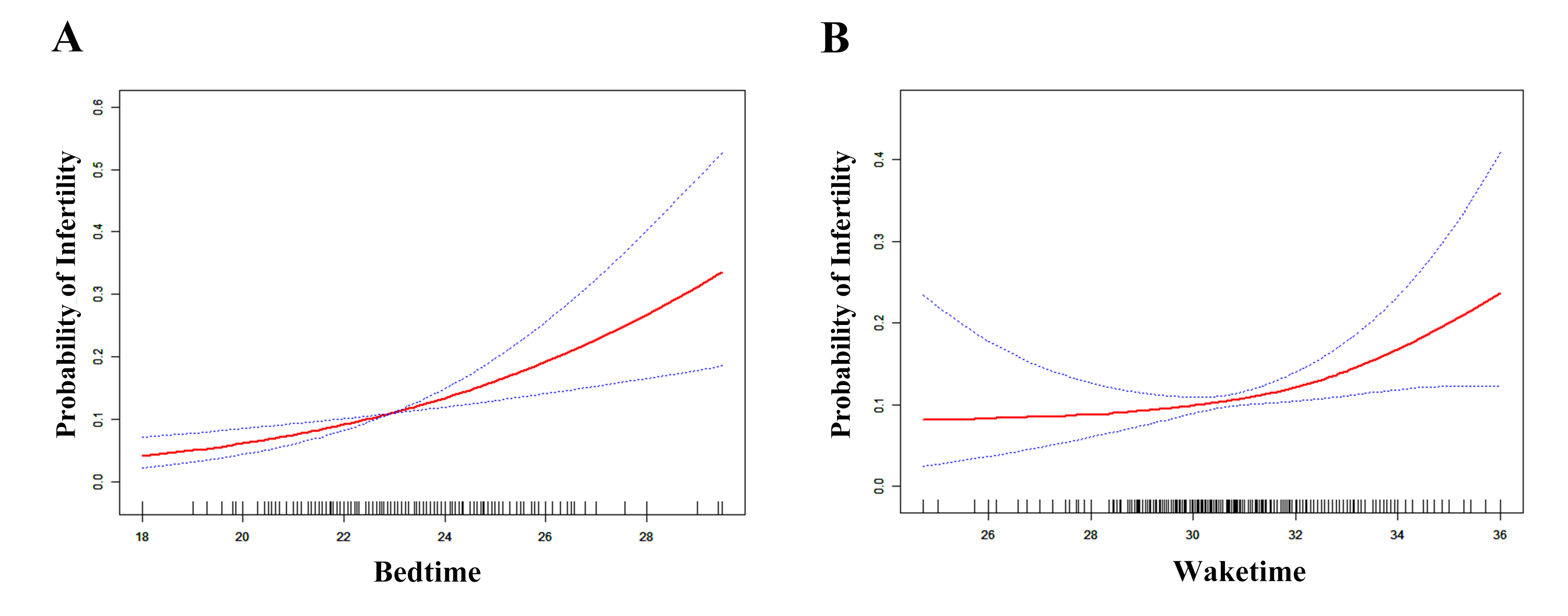

Supplement: Supplementary file 2 [file Image_2.tif]
