# Supplementary material for: Sleep Behavior and Self-Reported Infertility: A Cross-Sectional Analysis Among U.S. Women
Source: Front Endocrinol (Lausanne). 2022 May 10;13:818567. doi: 10.3389/fendo.2022.818567 (PMC9127231; doi:10.3389/fendo.2022.818567)
Supplement: Supplementary file 3 [file Table_1.docx]

**Table S1.** Multivariate analyses for infertility in unweighted sample (including bedtime).

| **Covariate** | **Full multivariate logistic regression** | | **AIC-based multivariate logistic regression** | |
| --- | --- | --- | --- | --- |
|  | **OR (95%CI)** | ***P*-value** | **OR (95%CI)** | ***P*-value** |
| **Bedtime** | 1.24 (1.10, 1.40) | **<0.001** | 1.19 (1.07, 1.33) | **0.001** |
| **Sleep duration (hours)** | 1.06 (0.94, 1.21) | 0.312 | / | / |
| **Age (years)** | 1.05 (1.03, 1.08) | **<0.001** | 1.05 (1.02, 1.07) | **<0.001** |
| **BMI (kg/m^2^)** | 1.03 (1.01, 1.04) | **0.004** | 1.03 (1.01, 1.04) | **0.003** |
| **Poverty level index (%)** |  |  |  |  |
| ≤ 1.30 | Reference |  | Reference |  |
| 1.30 - ≤ 1.85 | 0.60 (0.35, 1.02) | 0.060 | 0.60 (0.35, 1.01) | 0.056 |
| > 1.85 | 1.09 (0.75, 1.59) | 0.650 | 1.06 (0.75, 1.51) | 0.726 |
| **Physical activity (%)** |  |  |  |  |
| Yes | Reference |  | / |  |
| No | 1.04 (0.76, 1.44) | 0.793 | / | / |
| **Education (%)** |  |  |  |  |
| Less than high school | Reference |  | / |  |
| High school | 1.35 (0.79, 2.33) | 0.275 | / | / |
| More than high school | 1.12 (0.68, 1.85) | 0.645 | / | / |
| **Race (%)** |  |  |  |  |
| Mexican American | Reference |  | / |  |
| Other Hispanic | 0.69 (0.35, 1.28) | 0.223 | / | / |
| Non- Hispanic White | 0.88 (0.55, 1.40) | 0.577 | / | // |
| Non- Hispanic Black | 0.64 (0.37, 1.09) | 0.098 | / | / |
| Other Race | 0.80 (0.46, 1.38) | 0.423 | / | / |
| **Marital status (%)** |  |  |  |  |
| Married/Living with partner | Reference |  | Reference |  |
| Widowed/Divorced/Separated | 0.87 (0.56, 1.36) | 0.556 | 0.83 (0.54, 1.29) | 0.407 |
| Never Married | 0.42 (0.25, 0.71) | **0.001** | 0.39 (0.23, 0.66) | **<0.001** |
| **Cotinine (%)** |  |  |  |  |
| < 3 (ng/ml) (non-smoker) | Reference |  | Reference |  |
| ≥ 3 (ng/ml) (smoker) | 1.71 (1.17, 2.49) | **0.005** | 1.71 (1.19, 2.44) | **0.004** |

Note: OR = odds ratio; BMI = body mass index.

**Table S2.** Multivariate analyses for infertility in unweighted sample (including wake time).

| **Covariate** | **Full multivariate logistic regression** | | **AIC-based multivariate logistic regression** | |
| --- | --- | --- | --- | --- |
|  | **OR (95%CI)** | ***P*-value** | **OR (95%CI)** | ***P*-value** |
| **Wake time** | 1.14 (1.01, 1.28) | **0.037** | 1.13 (1.00, 1.27) | **0.042** |
| **Sleep duration (hours)** | 0.90 (0.79, 1.02) | 0.110 | 0.91 (0.81, 1.03) | 0.149 |
| **Age (years)** | 1.05 (1.02, 1.07) | **<0.001** | 1.05 (1.02, 1.07) | **<0.001** |
| **BMI (kg/m^2^)** | 1.03 (1.01, 1.05) | **0.004** | 1.03 (1.01, 1.05) | **0.002** |
| **Poverty level index (%)** |  |  |  |  |
| ≤ 1.30 | Reference |  |  |  |
| 1.30 - ≤ 1.85 | 0.61 (0.36, 1.04) | 0.070 | 0.61 (0.36, 1.03) | 0.064 |
| > 1.85 | 1.08 (0.74, 1.57) | 0.693 | 1.07 (0.76, 1.52) | 0.697 |
| **Physical activity (%)** |  |  |  |  |
| Yes | Reference |  |  |  |
| No | 1.05 (0.76, 1.45) | 0.757 |  |  |
| **Education (%)** |  |  |  |  |
| Less than high school | Reference |  |  |  |
| High school | 1.36 (0.79, 2.33) | 0.269 |  |  |
| More than high school | 1.12 (0.68, 1.84) | 0.663 |  |  |
| **Race (%)** |  |  |  |  |
| Mexican American | Reference |  |  |  |
| Other Hispanic | 0.69 (0.37, 1.32) | 0.260 |  |  |
| Non- Hispanic White | 0.89 (0.56, 1.42) | 0.630 |  |  |
| Non- Hispanic Black | 0.66 (0.39, 1.12) | 0.122 |  |  |
| Other Race | 0.84 (0.48, 1.45) | 0.533 |  |  |
| **Marital status (%)** |  |  |  |  |
| Married/Living with partner | Reference |  |  |  |
| Widowed/Divorced/Separated | 0.88 (0.57, 1.38) | 0.584 | 0.84 (0.54, 1.31) | 0.446 |
| Never Married | 0.42 (0.25, 0.71) | **0.001** | 0.40 (0.24, 0.66) | **<0.001** |
| **Cotinine (%)** |  |  |  |  |
| < 3 (ng/ml) (non-smoker) | Reference |  |  |  |
| ≥ 3 (ng/ml) (smoker) | 1.76 (1.21, 2.56) | **0.003** | 1.76 (1.23, 2.51) | **0.002** |

Note: OR = odds ratio; BMI = body mass index.

**Table S3.** Threshold effect analysis of wake time and infertility using piece-wise linear regression in unweighted sample.

| **Inflection point of wake time (hour)** | **Adjusted OR (95%CI)** | ***P*-value** |
| --- | --- | --- |
| **00:00 – < 08:00** | 1.00 (0.85, 1.19) | 0.970 |
| **08:00 - 12:00** | 1.41 (1.11, 1.79) | **0.004** |

Note: OR = odds ratio; BMI = body mass index.

Effect: infertility; Cause: wake time

***P* = 0.043 for** **logarithmic likelihood ratio test between wake time of 00:00 – < 08:00 and wake time of 08:00 - 12:00.**

Adjust for: age; race; BMI; education; marital status; physical activity; poverty level index; cotinine; sleep duration.

**Table S4.** Multivariate analyses for infertility in unweighted sample (including sleep behavior).

| **Covariate** | **Full multivariate logistic regression** | | **AIC-based multivariate logistic regression** | |
| --- | --- | --- | --- | --- |
|  | **OR (95%CI)** | ***P*-value** | **OR (95%CI)** | ***P*-value** |
| **Sleep behavior（categories）** |  |  |  |  |
| EE | Reference |  | Reference |  |
| EL | 2.02 (0.80, 5.07) | 0.137 | 2.12 (0.93, 4.84) | 0.074 |
| LE | 2.29 (1.52, 3.45) | **<0.001** | 2.11 (1.47, 3.03) | **<0.001** |
| LL | 2.14 (1.35, 3.41) | **0.001** | 2.09 (1.32, 3.32) | **0.002** |
| **Sleep duration (hours)** | 1.03 (0.89, 1.20) | 0.662 |  |  |
| **Age (years)** | 1.05 (1.03, 1.08) | **<0.001** | 1.05 (1.02, 1.07) | **<0.001** |
| **BMI (kg/m^2^)** | 1.03 (1.01, 1.04) | **0.005** | 1.03 (1.01, 1.04) | **0.003** |
| **Poverty level index (%)** |  |  |  |  |
| ≤ 1.30 | Reference |  |  |  |
| 1.30 - ≤ 1.85 | 0.62 (0.37, 1.06) | 0.081 | 0.62 (0.37, 1.05) | 0.077 |
| > 1.85 | 1.12 (0.77, 1.63) | 0.558 | 1.10 (0.78, 1.57) | 0.584 |
| **Physical activity (%)** |  |  |  |  |
| Yes | Reference |  |  |  |
| No | 1.05 (0.76, 1.44) | 0.789 |  |  |
| **Education (%)** |  |  |  |  |
| Less than high school | Reference |  |  |  |
| High school | 1.40 (0.81, 2.41) | 0.225 |  |  |
| More than high school | 1.13 (0.68, 1.86) | 0.635 |  |  |
| **Race (%)** |  |  |  |  |
| Mexican American | Reference |  |  |  |
| Other Hispanic | 0.66 (0.35, 1.27) | 0.216 |  |  |
| Non- Hispanic White | 0.88 (0.55, 1.41) | 0.596 |  |  |
| Non- Hispanic Black | 0.65 (0.38, 1.10) | 0.109 |  |  |
| Other Race | 0.78 (0.45, 1.35) | 0.367 |  |  |
| **Marital status (%)** |  |  |  |  |
| Married/Living with partner | Reference |  |  |  |
| Widowed/Divorced/Separated | 0.87 (0.56, 1.36) | 0.548 | 0.84 (0.54, 1.30) | 0.426 |
| Never Married | 0.41 (0.24, 0.69) | **0.001** | 0.39 (0.23, 0.64) | **<0.001** |
| **Cotinine (%)** |  |  |  |  |
| < 3 (ng/ml) (non-smoker) | Reference |  |  |  |
| ≥ 3 (ng/ml) (smoker) | 1.74 (1.19, 2.53) | **0.004** | 1.73 (1.21, 2.47) | **0.003** |

Note: EE = Early-bed/Early-rise; EL = Early-bed/Late-rise; LE = Late-bed/Early-rise; LL = Late-bed/Late-rise; OR = odds ratio; BMI = body mass index.

**Table S5.** Relationship between sleep behavior and infertility in unweighted sample.

| **Exposure** | **Unadjusted** | | **Adjusted*** | |
| --- | --- | --- | --- | --- |
|  | **OR (95%CI)** | ***P*-value** | **OR (95%CI)** | ***P*-value** |
| **Sleep behavior（categories）** |  |  |  |  |
| EE | Reference |  | Reference |  |
| EL | 1.12 (0.52, 2.39) | 0.780 | 2.02 (0.80, 5.07) | 0.137 |
| LE | 1.76 (1.27, 2.44) | **0.001** | 2.29 (1.52, 3.45) | **<0.001** |
| LL | 1.29 (0.86, 1.93) | 0.214 | 2.14 (1.35, 3.41) | **0.001** |
| **Sleep behavior (continuous）** | 1.16 (1.03, 1.30) | **0.017** | 1.34 (1.16, 1.53) | **<0.001** |

Note: EE = Early-bed/Early-rise; EL = Early-bed/Late-rise; LE = Late-bed/Early-rise; LL = Late-bed/Late-rise; OR = odds ratio; BMI = body mass index.

*Adjust for: age; race; BMI; education; marital status; physical activity; poverty level index; cotinine; sleep duration.

**Table S6.** Relationship between sleep behavior and infertility in weighted sample.

| **Exposure** | **Adjusted*** | |
| --- | --- | --- |
|  | **OR (95%CI)** | ***P*-value** |
| **Sleep behavior（categories）** |  |  |
| EE | Reference |  |
| EL | 2.00 (0.67, 5.93) | 0.237 |
| LE | 1.66 (1.07, 2.57) | **0.045** |
| LL | 1.61 (1.00, 2.60) | 0.076 |
| **Sleep behavior (continuous）** | 1.20 (1.04, 1.40) | **0.029** |

Note: EE = Early-bed/Early-rise; EL = Early-bed/Late-rise; LE = Late-bed/Early-rise; LL = Late-bed/Late-rise; OR = odds ratio; BMI = body mass index.

*Adjust for: age; race; BMI; education; marital status; physical activity; poverty level index; cotinine; sleep duration.

**Figure legends:**

**Figure S1. Adjusted associations of sleep duration with infertility in weighted sample.** Adjust for: age; race; BMI; education; marital status; physical activity; poverty level index; cotinine; sleep duration.

**Figure S2. Adjusted associations of bedtime (A), waketime (B) with infertility in weighted samples.** Adjust for: age; race; BMI; education; marital status; physical activity; poverty level index; cotinine; sleep duration.
